# Supplementary material for: A comparative whole-genome approach identifies bacterial traits for marine microbial interactions
Source: Commun Biol. 2022 Mar 28;5:276. doi: 10.1038/s42003-022-03184-4 (PMC8960797; doi:10.1038/s42003-022-03184-4)
Supplement: Supplementary file 3 — Description of Additional Supplementary Files [file 42003_2022_3184_MOESM3_ESM.pdf]

Description of Additional Supplementary Files

File name: Supplementary Data 1-11

Description:

Supplementary Data 1: Summary table of studies focused on genome clustering.

Supplementary Data 2: Table of interaction traits annotated in genomes.

Supplementary Data 3: Genomes' metadata table.

Supplementary Data 4: B vitamin strategies; source data for Figure 3.

Supplementary Data 5: Annotation table of B vitamins and siderophores.

Supplementary Data 6: Annotation table of KEGG modules.

Supplementary Data 7: Annotation table of secondary metabolites.

Supplementary Data 8: table of linked trait clusters (LTCs).

Supplementary Data 9: Annotation table of phytohormones.

Supplementary Data 10: Table of manually annotated traits.

Supplementary Data 11: Benchmark for KEGG modules' completeness thresholding.
