# Supplementary material for: A comparative whole-genome approach identifies bacterial traits for marine microbial interactions
Source: Commun Biol. 2022 Mar 28;5:276. doi: 10.1038/s42003-022-03184-4 (PMC8960797; doi:10.1038/s42003-022-03184-4)
Supplement: Supplementary file 5 — Reporting summary [file 42003_2022_3184_MOESM5_ESM.pdf]

Corresponding author(s): Luca Zoccarato
Last updated by author(s): Jun 23, 2021

Reporting Summary

Nature Research wishes to improve the reproducibility of the work that we publish. This form provides structure for consistency and transparency in reporting. For further information on Nature Research policies, see our Editorial Policies and the Editorial Policy Checklist.

Statistics

For all statistical analyses, confirm that the following items are present in the figure legend, table legend, main text, or Methods section.

n/a	Confirmed	
☐	☒	The exact sample size (*n*) for each experimental group/condition, given as a discrete number and unit of measurement
☒	☐	A statement on whether measurements were taken from distinct samples or whether the same sample was measured repeatedly
☐	☒	The statistical test(s) used AND whether they are one- or two-sided<br>*Only common tests should be described solely by name; describe more complex techniques in the Methods section.*
☐	☒	A description of all covariates tested
☐	☒	A description of any assumptions or corrections, such as tests of normality and adjustment for multiple comparisons
☐	☒	A full description of the statistical parameters including central tendency (e.g. means) or other basic estimates (e.g. regression coefficient) AND variation (e.g. standard deviation) or associated estimates of uncertainty (e.g. confidence intervals)
☐	☒	For null hypothesis testing, the test statistic (e.g. *F*, *t*, *r*) with confidence intervals, effect sizes, degrees of freedom and *P* value noted<br>*Give P values as exact values whenever suitable.*
☒	☐	For Bayesian analysis, information on the choice of priors and Markov chain Monte Carlo settings
☐	☒	For hierarchical and complex designs, identification of the appropriate level for tests and full reporting of outcomes
☐	☒	Estimates of effect sizes (e.g. Cohen's *d*, Pearson's *r*), indicating how they were calculated

Our web collection on statistics for biologists contains articles on many of the points above.

Software and code

Policy information about availability of computer code

Data collection	Genomes from NCBI were downloaded through FTP using wget command in a Linux terminal. Genomes from JGI were downloaded through the graphic interface available at https://img.jgi.doe.gov/cgi-bin/mer/main.cgi
Data analysis	Genome completeness was assessed using CheckM 1.0.11. The codes for functional annotation, reconstruction of KEGG modules and statistical analysis are available on GitHub (https://github.com/lucaz88/genome_comparison_code). Genome annotation was performed using Prokka 1.14.5, kofamscan 1.2.0 (using KEGG database v94.0), Anti-SMASH 5.1.2, BioV suite 1.0, NCBI blastp 2.10.0+. Plotting and statistical analyses have been performed in R 4.0.4 using the packages apcluster 1.4.8, circlize 0.4.13, clusterExperiment 2.12.0, ComplexHeatmap 2.8.0, ComplexUpset 1.3.0, dplyr 1.0.7, ggplot2 3.3.4, iheatmapr 0.5.1, indicspecies 1.7.9, reshape2 1.4.4, waffle 0.7.0.

For manuscripts utilizing custom algorithms or software that are central to the research but not yet described in published literature, software must be made available to editors and reviewers. We strongly encourage code deposition in a community repository (e.g. GitHub). See the Nature Research guidelines for submitting code & software for further information.

Data

Policy information about availability of data

All manuscripts must include a data availability statement. This statement should provide the following information, where applicable:

- Accession codes, unique identifiers, or web links for publicly available datasets
- A list of figures that have associated raw data
- A description of any restrictions on data availability

All genomes are available in the on-line repositories of NCBI and JGI under the accession codes listed in Supplementary table 3. The codes for functional annotation, reconstruction of KEGG modules and statistical analysis are available on GitHub (https://github.com/lucaz88/genome_comparison_code).

Field-specific reporting

Please select the one below that is the best fit for your research. If you are not sure, read the appropriate sections before making your selection.

☐ Life sciences ☐ Behavioural & social sciences ☒ Ecological, evolutionary & environmental sciences

For a reference copy of the document with all sections, see nature.com/documents/nr-reporting-summary-flat.pdf

Ecological, evolutionary & environmental sciences study design

All studies must disclose on these points even when the disclosure is negative.

Study description	The manuscript provides a first comprehensive atlas picturing the functional diversity of marine bacteria and their potential to interact with other microorganisms, based on a newly developed trait-based approach.
Research sample	The analyzed dataset included 473 complete genomes of bacteria. Of these 473 genomes, 421 were isolated in marine pelagic and coastal zones, 34 in extreme environments (e.g. salt marsh or hydrothermal vent), 6 in marine sediment and, of the remaining, 8 were human associated and 4 plant roots associated. The genomes represent a wide diversity of typical marine bacterial taxa and the sequenced genomes were available at NCBI and JGI data repositories under the accession number listed in Supplementary table 3.
Sampling strategy	We downloaded all complete genomes of bacteria that were freely available and for which the metadata could confirm a marine isolation source.
Data collection	Genomes were manually downloaded from the repositories or using a Linux bash script (wget).
Timing and spatial scale	Genomes were retrieve from online repositories during 2018.
Data exclusions	No data was excluded from the analysis.
Reproducibility	It's a bioinformatic manuscript and all analyses are fully reproducible with the provided information.
Randomization	Genomes were clustered into coherent functional groups using a machine learning algorithm that identifies the best cluster configuration. It works in an iterative fashion where at each instance a random set of genomes is picked as seeds for creating the clusters.<br>Genetic traits are grouped into linked trait clusters by mean of an automatic function "cutreeDynamic". This function extracts the clusters from a hierarchical clustering of the dissimilarity matrix built on pairwise Pearson's correlation coefficient (r2) between each genetic traits.
Blinding	Not applicable due to the nature of the data (genome sequences).

Did the study involve field work? ☐ Yes ☒ No

Reporting for specific materials, systems and methods

We require information from authors about some types of materials, experimental systems and methods used in many studies. Here, indicate whether each material, system or method listed is relevant to your study. If you are not sure if a list item applies to your research, read the appropriate section before selecting a response.

Materials & experimental systems

n/a	Involved in the study
☒	☐ Antibodies
☒	☐ Eukaryotic cell lines
☒	☐ Palaeontology and archaeology
☒	☐ Animals and other organisms
☒	☐ Human research participants
☒	☐ Clinical data
☒	☐ Dual use research of concern

Methods

n/a	Involved in the study
☒	☐ ChIP-seq
☒	☐ Flow cytometry
☒	☐ MRI-based neuroimaging
